# Immune Activation in Primary Sclerosing Cholangitis: A Systematic Review and Comparative Analysis With Inflammatory Bowel Diseases

**DOI:** 10.1002/ueg2.70115

**Published:** 2025-09-30

**Authors:** Md Moniruzzaman, Ayesha Shah, Thomas Fairlie, Simon Keely, Grace L. Burns, Nicholas Talley, Gerald Holtmann

**Affiliations:** ^1^ Faculty of Health, Medicine, and Behavioural Sciences, The University of Queensland, Translational Research Institute Brisbane Australia; ^2^ Frazer Institute, The University of Queensland Brisbane Australia; ^3^ Department of Gastroenterology & Hepatology, Princess Alexandra Hospital Brisbane Australia; ^4^ School of Biomedical Sciences & Pharmacy, College of Health Medicine and Wellbeing, University of Newcastle Newcastle Australia; ^5^ Immune Health Research Program, Hunter Medical Research Institute, New Lambton Heights Newcastle Australia; ^6^ Centre of Research Excellence in Digestive Health, University of Newcastle Newcastle Australia; ^7^ School of Medicine & Public Health, College of Health Medicine and Wellbeing, University of Newcastle Newcastle Australia

**Keywords:** cytokines, immune activation, inflammatory bowel diseases, primary sclerosing cholangitis, regulatory T cells (Tregs), Th17 cells, ulcerative colitis

## Abstract

**Background and Objectives:**

Primary sclerosing cholangitis (PSC) is a chronic liver disease with aberrant immune dysregulation and bile duct fibrosis. It is often associated with inflammatory bowel disease (IBD), especially ulcerative colitis, raising questions about distinct immune activation in these conditions. Therefore, we aimed to systematically review and compare immune activation patterns in patients with PSC and IBD (without PSC), which may provide deeper insights into PSC pathophysiology.

**Methods:**

MEDLINE, Scopus, Cochrane Library, and Embase were searched until July 2024 for relevant studies reporting immune cell profiles, cytokine levels, and gene expression patterns in patients with PSC. Reference articles of patients with IBD were then added to compare the immune profile of patients with PSC (with or without IBD) and patients with IBD‐only.

**Results:**

Twenty‐three articles studying 638 PSC and 557 non‐PSC non‐IBD subjects were included. PSC patients showed various degrees of immune activation in the systemic circulation, biliary fluid, and liver tissue, most notably regarding integrin β7+ gut‐homing T cells, IL‐2, and IL‐10 compared to their respective controls. Compared with patients with IBD, patients with PSC had reduced Tregs in the systemic circulation. When comparing tissue‐based immune markers, PSC‐livers had increased Th17 cells, IL‐1β, and TNF‐α and reduced levels of B cells, IL‐2, and IL‐10 than the IBD‐mucosa.

**Conclusions:**

Patients with PSC and patients with IBD without PSC can be differentiated by a distinct immune activation pattern with upregulation of Th17 and downregulation of Treg functions in PSC while other immune parameters do not allow a differentiation of these conditions.

## Summary

- Established knowledge of the subject:
  ◦ Primary sclerosing cholangitis (PSC) involves aberrant activation of innate and adaptive immune responses leading to chronic inflammation and fibrosis in the liver and bile ducts.
  ◦ Inflammatory bowel diseases (IBD), especially ulcerative colitis (UC) often co‐exist with PSC, which may complicate the immunological landscape in patients with PSC.
- Significant and/or new findings of this study:
  ◦ The immune activation profiles of the systemic circulation, liver tissue, and biliary fluid of the patients with PSC are different.
  ◦ Despite the similarities with IBD, patients with PSC possess increased Th17 and reduced regulatory immune responses, which may be different from IBD alone.

## Graphical abstract

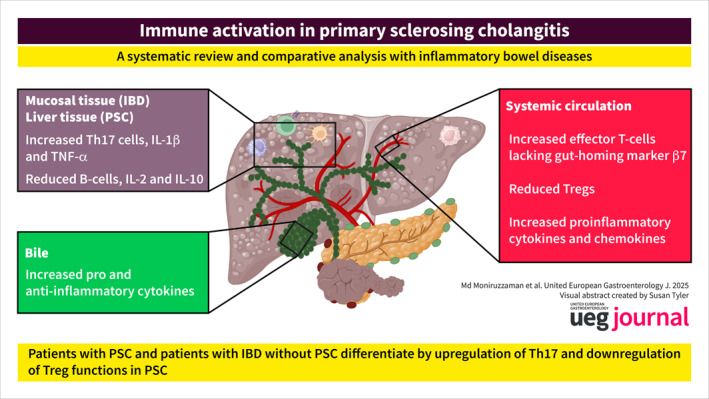

**Abbreviations**
β7: gut‐homing integrin β7
CCL: Chemokine (C‐C motif) ligand
CCR6: C‐C chemokine receptor type 6
CD: Crohn’s disease
CD: cluster of differentiation
CXCL: Chemokine (C‐X‐C motif) ligand
IBD: inflammatory bowel diseases
IFN: interferon
IL: interleukin
NK: natural killer cells
PBC: primary biliary cholangitis
PBMCs: peripheral blood mononuclear cells
PSC: primary sclerosing cholangitis
Tfh: T follicular helper cell
Th: T‐helper cell
TNF: tumour necrosis factor
Treg: regulatory T cells
UC: ulcerative colitis

## 1 Introduction

Primary sclerosing cholangitis (PSC) is a rare chronic cholestatic liver disease characterised by inflammation and fibrosis of both intrahepatic and extrahepatic bile ducts. These lead to the formation of multifocal bile duct strictures and progressive fibrotic transformation of bile ducts [1], resulting in over 10–15 years of end‐stage liver disease, ultimately requiring liver transplantation for treatment [1]. PSC is closely associated and often concomitant with inflammatory bowel diseases (IBD) [2], especially ulcerative colitis (UC). Furthermore, PSC‐UC patients are characterised by a unique clinical presentation of a right‐sided phenotype in terms of disease location and activity, as well as a high rate of gastrointestinal and hepatobiliary malignancies [3, 4].

IBD is well documented to have dysregulated immune responses [5, 6]; however, PSC data explaining the immunological mechanisms of the disease are limited. Previous studies have shown that autoantibodies, gut‐homing T cells, and susceptibility and resistance to human leucocyte antigen contributed to the peribiliary and fibrosing inflammation seen in PSC [7, 8]. In PSC, activated cholangiocytes may facilitate the retention and homing of gut‐homing T cells in the liver, initiating uncontrolled immune activation [7, 8, 9]. This leads to chronic inflammation and fibrosis in the liver and bile ducts, causing restricted transport of bile from the liver to the small intestine [10]. Intestinal dysbiosis may further drive immune‐mediated liver damage in patients with PSC [11]. The persistent and uncontrolled cascade of sequential events leads to liver cirrhosis and increases the risk of hepatobiliary cancer [12].

Given the complexity of PSC and the lack of established efficacious medical therapies [13], research is ongoing to understand the pathobiological mechanisms and develop effective therapies by targeting the immune system [14], microbiome [15], and fibrosis [16]. While a complete immune profile in patients with PSC is missing, immune dysregulation is a common feature in both PSC and IBD. Given the concomitant nature of the two conditions [17], an opportunity exists to explore differences and similarities in the immune processes linked to the clinical manifestation of PSC and IBD to better understand disease pathophysiology. This ultimately may guide future therapeutic approaches in patients with PSC and IBD. Against this background, this systematic review aims to understand the level of immune activation in patients with PSC by assessing available data and specifically defining differences in the immune pathophysiology of patients with PSC (with or without IBD) and IBD without PSC.

## 2 Materials and Methods

### 2.1 Protocol and Registration

This systematic review meets the preferred reporting items for systematic reviews and meta‐analysis statement requirements (PRISMA) [18, 19, 20]. The protocol for this Systematic Review was prospectively registered with PROSPERO (CRD42024596722).

### 2.2 Search Strategy

**FIGURE 1:**
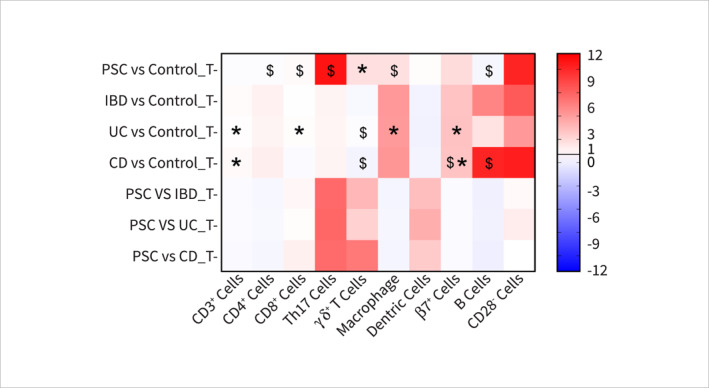
Heatmap of the fold change (FC) of tissue‐based immune cell markers in patients with PSC, IBD, UC, or CD. All FC values are > 0, where FC > 1 (shown in red) indicates upregulation and FC < 1 (> 0 and shown in blue) indicates downregulation. In *Y*‐axis, PSC: primary sclerosing cholangitis, IBD: inflammatory bowel diseases, UC: ulcerative colitis, CD: Crohn's disease, T: tissue‐based results, *inconsistent findings across studies, $: results with low confidence from studies with low methodological scores. In *X*‐axis, CD: cluster of differentiation, Th: T‐helper cell, and β7: gut‐homing integrin β7.

We searched electronic databases, including MEDLINE, Scopus, Cochrane Library, and Embase, from inception until July 2024. The literature search strategy is outlined in the PRISMA flow diagram (Supporting Information S1: Figure 1) and was conducted with the assistance of our librarian. The search strategy has been outlined in Supporting Information S1: Table 1. The search initially focused on human studies and did not impose any restrictions on specific languages in order to include all relevant studies. A further advanced search was conducted. Grey literature was searched with Google and Google Scholar, and the ‘Snowball’ method was also utilised to identify all relevant articles. Literature describing each immune parameter in UC and Crohn's disease (CD) was also searched separately in the above‐mentioned databases and incorporated as a reference for comparative analysis (Supporting Information S1: Table 2).

### 2.3 Study Selection

Two authors (M.M. and T.F.) independently screened abstracts and titles. All in vivo articles were excluded from the study. Abstracts were also excluded if the study did not investigate the association of immune activation in PSC and PSC‐IBD. Full texts of the remaining articles, including conference abstracts, were retrieved and reviewed. The inclusion criteria include human studies assessing immune activation in blood, peripheral blood mononuclear cells (PBMCs), serum, plasma, liver samples, and biliary fluid to identify changes in any immune cell subsets and/or inflammatory mediators in patients with PSC (with or without IBD) in comparison to the non‐PSC non‐IBD subjects.

### 2.4 Data Extraction

Data were extracted independently by two authors (M.M. and T.F.), with discrepancies resolved by reference to the source publication. Data were entered into a Microsoft Excel spreadsheet (Office 365: Microsoft Corp, Redmond, Washington, USA). The data extracted include demographic details (age, gender, and study location), clinical data (presence and type of IBD, medications, types of controls and types of samples analysed), and immune data (subtypes of immune cells and levels of cytokines and chemokines in the systemic circulation, liver, and/or biliary fluid measured using flow cytometry, enzyme linked immunosorbent assay, western blotting, histology and quantitative polymerase chain reaction).

### 2.5 Quality Assessment

Generally, a set of assessment criteria is designed to evaluate the cross‐sectional and randomised control studies, which is not well‐suited for quantitative observational studies consisting of a range of experimental methodologies and populations [21]. Therefore, to properly fit our review, we modified the article assessment criteria by Burns et al. [22] by designing a 3‐point score (0, 1, and 2) for a total of 10 criteria (Supporting Information S1: Table 3), which also comply with the quality assessment tools for systematic review of in vitro studies [23]. Then, the articles were assessed against a possible maximum score of 20.

### 2.6 Data Analysis

Fold changes in immune markers were calculated by dividing the values in PSC or IBD with values of their respective controls. These fold changes were labelled as PSC, IBD, UC, or CD for individual sample types (circulation ‘C’, tissue ‘T’, and ‘Bile’). To compare PSC with IBD, the PSC ratios were divided by the IBD ratios. Subsequently, the values were plotted in GraphPad Prism version 10 (Boston, MA, USA) to generate a heatmap for visualisation.

## 3 Results

### 3.1 Selection Outcome

The initial literature search revealed 6648 publications. Of these, 127 published articles were relevant to the study question and were retrieved for further evaluation. Of these, 104 were excluded for various reasons, leaving 23 eligible studies (Supporting Information S1: Figure 1) involving 638 PSC (345 with concomitant IBD) and 557 non‐PSC non‐IBD subjects. As the majority of these studies did not differentiate the immune status in patients with PSC only from PSC‐IBD (UC or CD), therefore, we considered these two cohorts under PSC and compared the cumulative immune signatures with the IBD and subgroups (Table 1). Additionally, a subset of included studies (5 out of 23) used non‐PSC non‐IBD diseased control, for instance, patients with PBC, ALD, HepC, SSC, AC, and other chronic liver conditions as a control instead of healthy subjects. This variability in control group selection was taken into consideration during the methodological quality assessment (criteria 3), as different baseline immune profiles may confound interpretation. PBC subjects may indeed have a distinct immune profile from the healthy individuals. However, Hashimoto et al. 1993 reported a clear and significant difference in the population of CD11b+ macrophages (2.4% vs. 6.1%) and CD56+ NK cells (2.9% vs. 6.0%) between patients with PBC and PSC [36]. While we considered a sub‐analysis comparing PSC to non‐immune‐related liver diseases, it did not substantially alter the key trends observed in immune cell/cytokine alterations. For instance, increased γδ+ T cells in the liver tissue [32] and IL‐6 and IL‐8 in the systemic circulation [45] support the consistency of our findings (data not shown). In addition, some observations such as increase of Th17 cells in liver tissue [38], γδ+ T cells [32] and TNF‐α in the systemic circulation, IL‐8 [45], IL‐2, IL‐4, IL‐6, IL‐10, IL‐17A, and IFN‐γ [46] in the biliary fluid were reported exclusively by these studies and are therefore interpreted with caution.

**TABLE 1:** Patient demographics of the included PSC studies.

No	Author	Study year	Journal	Region	Quality score (/20)	Sample type	Patients with PSC (*N*)	IBD in patients with PSC (*N*)	Male (%)	Mean or median age (years)	With medication (%)	Controls (*N*)	Type of control	Male (%)	Mean or median age (years)
1	Abarbanel et al. [24]	2013	J Clin Immunol	United States	13	PBMCs	8	8	NA	11.7 ± 5.3	100	9	Healthy control	NA	11.7 ± 5.3
2	Adam et al. [25]	2018	Hepatol Commun	Germany	15	PBMCs	20	16	65	44.6 ± 15.1	100	23	Healthy control	0	NA
3	Bo et al. [26]	2001	Gut	Sweden	10	PBMCs	7	5	NA	42 (36–63)	100	8	Healthy control	NA	NA
4	Broome et al. [27]	1998	Dis Colon Rectum	Sweden	14	PBMCs	11	11	45.45	NA	0	5	Healthy control	60	NA
5	Gwela et al. [28]	2017	J Crohns Colitis	United Kingdom	14	PBMCs	31	31	62	52 (25–75)	62	34	Healthy control	47	62 (26–91)
6	Dold et al. [29]	2023	Clin Transl Gastroenterol	Germany	16	PBMCs	50	36	56	41 (20–67)	92	36	Healthy control	35.78	41 (20–79)
7	Katt et al. [30]	2013	Hepatology	Germany	14	PBMCs	46	46	82.6	40 (19–71)	NA	26	Healthy control	NA	NA
8	Liu et al. [31]	2022	Front Immunol	United States	11	PBMCs	45	NA	47.73	NA	NA	44	Healthy control	52.23	NA
9	Martins et al. [32]	1996	Hepatology	United Kingdom	10	PBMCs	25	2	64	55	8	54	Healthy, ALD, HepC	48.15	NA
10	Poch et al. [33]	2021	J Hepatol	Germany	13	PBMCs	8	0	75	49 ± 3	0	10	Healthy control	50	42.5 ± 15.5
11	Sebode et al. [34]	2014	J Hepatol	Germany	14	PBMCs	47	∼27	∼76.62	42 (19–75)	∼79.22	44	Healthy control	∼46.67	31 (22–68)
3	Bo et al. [26]	2001	Gut	Sweden	10	Liver	7	5	NA	42 (36–63)	NA	8	Healthy control	NA	NA
12	Graham et al. [35]	2022	Hepatology	United Kingdom	12	Liver	7	7	100	NA	NA	4	Healthy control	NA	NA
13	Hashimoto et al. [36]	1993	Mayo Clin Proc	Japan	7	Liver	19	NA	47.37	36	0	20	Patients with PBC	0	48.33
14	Liaskou et al. [37]	2014	Gastroenterology	United Kingdom	10	Liver	11	NA	NA	NA	NA	4	Healthy control	NA	NA
9	Martins et al. [32]	1996	Hepatology	United Kingdom	10	Liver	14	NA	NA	NA	NA	26	Healthy, ALD, HepC	NA	NA
15	Tedesco et al. [38]	2018	Gastroenterology	Finland	7	Liver	5	NA	NA	NA	NA	5	Patients with HepC	NA	NA
16	Bansal et al. [39]	1997	Autoimmunity	Australia	8	Serum	31	23	45.16	42 (15–59)	NA	16	Healthy control	45	45 (21–59)
17	Dhillon et al. [40]	2019	Liver Int	Norway	16	Serum	166	121	79.5	41 (16.3–72.4)	83.34	100	Healthy control	NA	NA
6	Dold et al. [29]	2023	Clin Transl Gastroenterol	Germany	16	Serum	50	36	56	41 (20–67)	100	36	Healthy control	35.78	41 (20–79)
18	Lampinen et al. [41]	2018	J Leukoc Biol	Germany	15	Serum	6	6	83.33	44.0 (30–65)	100	19	Healthy control	42.1	47.6 (24–64)
19	Lampinen et al. [42]	2019	J Crohns Colitis	Germany	15	Serum	6	6	83.33	44.0 (30–65)	100	19	Healthy control	42.1	47.6 (24–64)
20	Landi et al. [43]	2014	J Interferon Cytokine Res	Germany	11	Serum	58	NA	67.24	46.2–11.1	0	50	Healthy control	54	50.3–8.2
21	Langeneckert et al. [44]	2019	Eur J Immunol	Germany	11	Plasma	10	NA	NA	NA	NA	10	Healthy control	NA	NA
22	Zweers et al. [45]	2016	Liver Int	Norway	10	Biliary fluid	13	NA	61.54	43 (25–70)	NA	11	Patients with AC, AIH, CC, HC, AIPHC	63.64	63 (42–78)
23	Zhou et al. [46]	2020	Eur J Gastroenterol Hepatol	Germany	12	Biliary fluid	8	NA	NA	NA	NA	15	Patients with SSC	NA	NA

*Note:* ∼ the approximate number was calculated from the percentage of patients provided.

Abbreviations: AC, alcoholic cirrhosis; AIH, auto‐immune hepatitis; AIPHC, acute intermittent porphyria with hepatocellular carcinoma; ALD, alcoholic liver disease; CC, cryptogenic cirrhosis; HC, haemochromatosis; HepC, hepatitis C; PBC, primary biliary cholangitis; PBMCs, Peripheral blood mononuclear cells; SSC, secondary sclerosing cholangitis.

We also found 59 articles differentially reporting the immune markers in the patients with IBD (UC and CD), where at least 42 studies used samples from patients with UC and 44 used CD (Supporting Information S1: Table 2). Most of these studies investigating immune activation in IBD systemic circulation used blood samples from healthy volunteers as controls. However, considering the challenges in obtaining colonic biopsies from healthy volunteers, those who analysed immune activation in colonic tissues mostly used control samples from non‐inflamed colon of the same IBD patients, patients with functional GI diseases, or those who participated in the colorectal cancer screening programme. Despite the diversity of the control groups, all studies showed significant differences in immune parameters in the IBD group, and therefore, these studies were used for comparisons of the immune markers with the patients with PSC.

### 3.2 Quality Assessment of Studies Included in This Systematic Review

The methodological quality assessment using the 3‐point scale revealed an average score of 12.04 (out of 20), with scores ranging from 7 to 16. While several low‐scored studies were included to extract the undisputed immune data (Table 1 and Supporting Information S1: Table 4), the diversity in quality scores provides valuable insights into the overall methodological landscape of the relevant literature in our review. None of these studies performed power analysis to identify the required number of samples for their analysis. We also observed that 9 out of the 23 studies involved 10 or fewer PSC subjects, potentially limiting the generalisability of their findings.

In addition, three studies with less than 10 quality scores were also added to the analysis, which includes Hashimoto et al. 1993 [36] (score: 8) reporting increased CD8+ T cells, macrophages and NK cells and decreased CD3+ and CD4+ T cells in the liver; Tedesco et al. 2018 [38] (score: 5) reporting increased Th17 and decreased γδ+ T cells in the liver tissue; and Bansal et al. 1997 [39] (score: 8) reporting increased IL‐8 and IL‐10 in the systemic circulation. Despite the low methodological quality scores, these articles were included in the analysis due to a limited number of available studies investigating immune activation in PSCs.

To assess the robustness of our findings, we performed a sensitivity analysis including only studies with a methodological quality score of ≥ 10 (out of 20). While this reduced the number of included studies (20 out of 23), the overall trends in immune cell activation and cytokine dysregulation affected by the low‐scored studies, for example, increase of IL‐8 and IL‐10 in systemic circulation and decrease of CD3+ T cells in the liver tissue remained consistent with the primary analysis, supporting the reliability of these observations. Some specific findings exclusively reported in the low‐scored studies (e.g., decrease of CD4+ T and B cells, increase of macrophages, CD8+ T [36], and Th17 cells [38] in the liver) were not replicated in higher‐quality studies, indicating a lower confidence in those results. In addition, due to the limited availability of immune data, we also included 5 (out of 59) IBD studies with quality scores less than 10 (out of 20). Therefore, all interpretations concluded solely from the low‐scored studies possessing a lower degree of confidence are highlighted in Figure 1.

### 3.3 Circulating Immune Cell Populations

**FIGURE 2:**
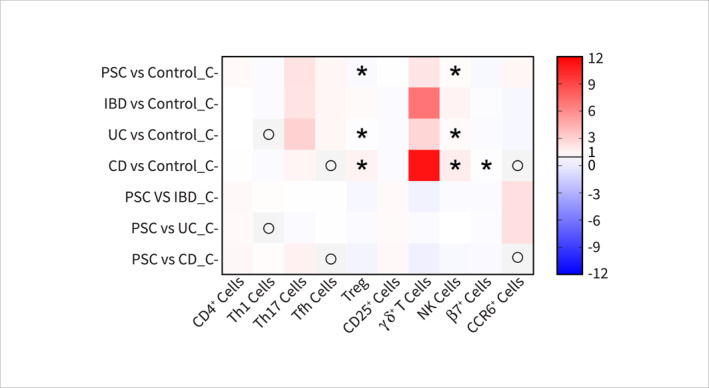
Heatmap of the fold change (FC) of systemic immune cell markers in patients with PSC, IBD, UC, or CD. All FC values are > 0, where FC > 1 (shown in red) indicates upregulation and FC < 1 (> 0 and shown in blue) indicates downregulation. In *Y*‐axis, PSC: primary sclerosing cholangitis, IBD: inflammatory bowel diseases, UC: ulcerative colitis, CD: Crohn's disease, C: systemic circulation (blood), ο: No available data, *inconsistent findings across studies. In *X*‐axis, CD: cluster of differentiation, Th: T‐helper cell, Tfh: T follicular helper cell, Treg: regulatory T cell, NK: natural killer cell, β7: gut‐homing integrin β7, and CCR6: C‐C chemokine receptor type 6.

**FIGURE 3:**
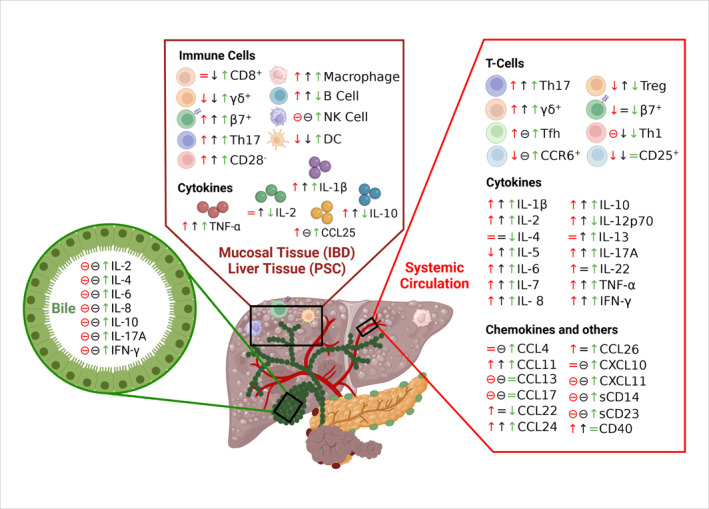
Comparison of immune parameters in patients with PSC, UC, and CD. Systemic circulation, liver tissue, and biliary fluid showed increased and decreased immune cell populations and cytokines. Th: T‐helper cell, Tfh: T follicular helper cell, Treg: regulatory T cells, NK: natural killer cells, DC: dendritic cell, β7: gut‐homing integrin β7, CD: cluster of differentiation, IL: interleukin, IFN: interferon, TNF: tumour necrosis factor, CCL: Chemokine (C‐C motif) ligand, and CXCL: Chemokine (C‐X‐C motif) ligand. Red: UC, black: CD, green: PSC, ↑: Increased, ↓: Decreased, =: No change, *⊖*: data not available. Figure was drawn with the help of Bio Render software.

Overall, 18 studies were included in this systematic review, assessing various subsets of immune cells and different inflammatory cytokines, chemokines, and other factors within the systemic circulation of patients with PSC. These include analysis of peripheral blood mononuclear cells (PBMCs), serum, and plasma isolated from the patients (Figures 2 and 3).

Out of the 18 studies, six [25, 28, 29, 30, 32, 33] reported an increase in various immune cell populations including CD4+ T [25], Th17 [25, 28, 29, 30, 33], Tfh [25], γδ+ T [32], and CCR6+ T cells [28] in the circulation compared to the control group (Figures 1 and 2 and Supporting Information S1: Table 4). On the other hand, four studies reported decreases in Th1 [25, 28], regulatory T cells (Treg) [24, 25, 34], and gut‐homing integrin β7+ T cells [28] cell populations in the circulation of patients with PSC. Additionally, the levels of CD3+ T [25, 26], Th2 [25], CD25+ T [26], and NK cells [26, 31] showed no significant change (Figure 2 and Supporting Information S1: Table 4). It is important to note that the reported levels of some of the markers, for instance, Treg and NK cells, were contested across the studies, which may require further investigation.

These findings align closely with the immune cell population changes observed in the systemic circulation of patients with UC and CD compared with their respective controls. The only exception was the level of chemoattractant receptor CCR6+ cells, which was lower in patients with UC compared with patients with PSC [28, 47]. The levels of CD25+ cells are also lower in both UC and CD compared to PSC patients [26, 48, 49]. Interestingly, the Treg population was found to be higher in patients with CD [50], while lower levels were observed in patients with both UC and PSC [24, 25, 34, 50]. PSC patients had a lower level of Tregs compared to those with UC (Figures 1 and 2) [24, 25, 34, 50]. Moreover, compared with UC and CD, PSC patients had a lower level of γδ+ T cells in the systemic circulation [32, 51].

### 3.4 Circulating Cytokines and Chemokines

**FIGURE 4:**
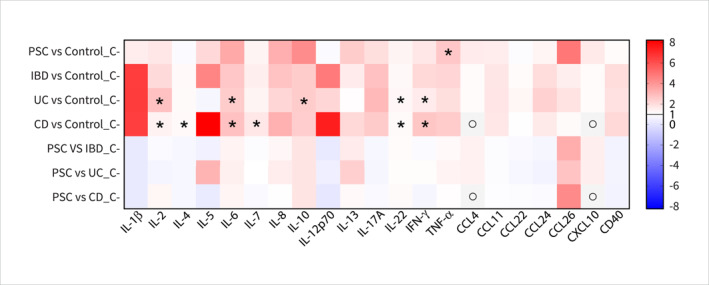
Heatmap of the fold change (FC) of systemic cytokines, chemokines, and soluble factors comparing patients with PSC, IBD, UC, or CD and controls. All FC values are > 0, where FC > 1 (shown in red) indicates upregulation and FC < 1 (> 0 and shown in blue) indicates downregulation. In *Y*‐axis, PSC: primary sclerosing cholangitis, IBD: inflammatory bowel diseases, UC: ulcerative colitis, CD: Crohn's disease, C: systemic circulation (blood), ο: No available data, *inconsistent findings across studies. In *X*‐axis, IL: interleukin, IFN: interferon, TNF: tumour necrosis factor, CCL: Chemokine (C‐C motif) ligand, CXCL: Chemokine (C‐X‐C motif) ligand, and CD: cluster of differentiation.

Ten studies [27, 29, 33, 39, 40, 41, 42, 43, 44, 45] provided comprehensive data on the levels of cytokines and chemokines in the systemic circulation in patients with PSC and controls (Figures 3 and 4 and Supporting Information S1: Table 4). A consensus increases in specific cytokines, chemokines, and soluble factors compared with their respective controls was reported. These include IL‐1β [43], IL‐2 [27, 43], IL‐5 [41, 43], IL‐6 [29, 43, 45], IL‐8 [39, 43, 45], IL‐10 [39, 43], IL‐13 [41, 43], IL‐17A [29], IL‐22 [33], IFN‐γ [29, 43], TNF‐α [43, 45], chemokines CCL4 [43], CCL11 or eotaxin‐1, CCL26 or eotaxin‐3 [41], CXCL10 or IP‐10 [43, 44], CXCL11 [44], and soluble factors CD14 [40] and CD23 [39] (Figure 4). Compared to controls, other markers such as IL‐4 [27, 43], IL‐7 [43], IL‐12p70 [43], IL‐18R1 [42], CCL13 or MCP‐4, CCL17 or TARC, CCL22 or MDC [43], CCL24 or eotaxin‐2 [41], CXCL9 [43], and soluble factor CD40 [42] remained relatively unchanged (Figures 3 and 4 and Supporting Information S1: Table 4). However, the levels of TNF‐α were contested in the reported studies. In line with the changes observed in immune cell populations, the levels of cytokines and chemokines in the circulation of patients with UC and CD also exhibited a similar trend (Figure 4).

However, compared to the patients with UC and CD, PSC patients exhibited slightly higher level of IL‐6, IL‐10, TNF‐α [52, 53], IL‐13 [41, 43, 52, 53], IL‐22 [33, 54], and CCL26 [41, 52, 55] and lower level of IL‐1β [43, 52, 53], IL‐4 [27, 43, 52, 53], IL‐12p70 [43, 52, 53], IL‐17A [29, 53], CCL22 [43, 52], CCL24 [41, 55], and CD40 [42, 56] and (Figures 3 and 4). Interestingly, while the patients with PSC exhibited lower levels of IL‐5 compared to those with CD, this level was higher than those in the UC cohort (Figure 4) [41, 43, 52, 53].

### 3.5 Liver Tissue Immune Cells and Cytokines

**FIGURE 5:**
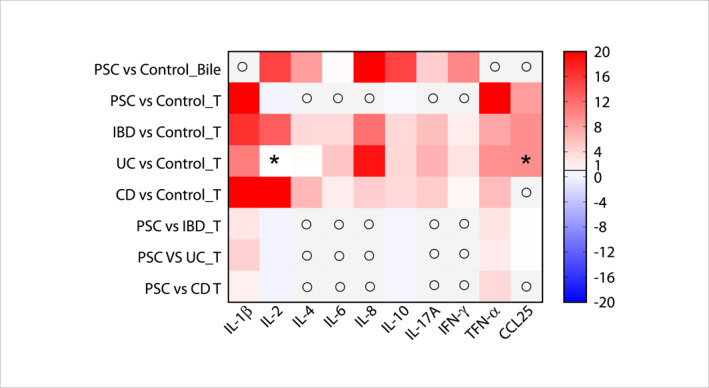
Heatmap of the fold change (FC) of biliary and tissue‐based cytokines, chemokines, and soluble factors comparing patients with PSC, IBD, UC, or CD and controls. All FC values are > 0, where FC > 1 (shown in red) indicates upregulation and FC < 1 (> 0 and shown in blue) indicates downregulation. In *Y*‐axis, PSC: primary sclerosing cholangitis, IBD: inflammatory bowel diseases, UC: ulcerative colitis, CD: Crohn's disease, T: tissue‐based results, ο: No available data, *inconsistent findings across studies. In *X*‐axis, IL: interleukin, IFN: interferon, TNF: tumour necrosis factor, and CCL: Chemokine (C‐C motif) ligand.

Six [26, 32, 35, 36, 37, 38] out of 23 studies specifically investigated subsets of immune cells, cytokines produced, and their changes within the liver tissue. They reported an increase in cytotoxic CD8+ T cells, macrophages [36], CD28‐ T cells [37], Th17 cells [38], γδ+ T cells [32, 38], NK cells [26, 36], gut‐homing β7+ T cells [35] (Figure 1 and Supporting Information S1: Table 4), and an increase in cytokines such as IL‐1β, TNF‐α [26], and chemokine CCL25 [35] (Figure 5 and Supporting Information S1: Table 4).

On the other hand, certain immune cell populations and cytokines were found to be reduced in the liver tissue in patients with PSC. These include CD3+ cells [26, 36], CD4+ cells, B cells [36] and cytokines IL‐10 and IL‐2 [26]. Interestingly, conflicting findings were reported for CD25+ regulatory T cells, with an increase reported by Hashimoto et al. and a reduction reported by Bo et al. [26, 36]. Similarly, the levels of NK cells and γδ+ T cells were also contested in reported studies. However, the overall levels of CD25‐expressing T cells [26, 36] and dendritic cells [36] remained unchanged in the liver of the patients with PSC (Figure 1 and Supporting Information S1: Table 4). Despite some similarities, this profile differs slightly from that seen in patients with IBD. Patients with CD were reported to have decreased populations of CD8+ T cells, whereas the level was almost unchanged in the UC patients [57]. Dendritic cells (DC) [36, 58] and γδ+ T cells [32, 59] were found to be decreased and B cells [36, 60] (Figure 1) and IL‐10 [26, 52, 61] (Figure 5) to be higher in both patient cohort of UC and CD compared to their respective control and liver tissue from the patients with PSC.

Although all other immune cell types and cytokines were increased in UC, CD, and PSC, the levels of Th17 cells [38, 62], IL‐1β [26, 52, 63], and TNF‐α [26, 52, 64] were higher in the PSC liver than in the inflamed intestine in UC and CD. On the other hand, macrophages [36, 58] and gut‐homing β7+ T cells [35, 65] in both UC and CD intestines had a higher expression level than the PSC liver tissue.

### 3.6 Cytokines in the Biliary Fluid

Two studies [45, 46] reported an increase in various cytokines in the biliary fluid of patients with PSC compared with the control group. These cytokines include IL‐2, IL‐4, IL‐6, IL‐10, IL‐17A, IFN‐γ [46], and IL‐8 [45] (Figure 4 and Supporting Information S1: Table 4). Importantly, the divergent immune responses observed in patients with PSC are highlighted by the contrasting levels of IL‐2 and IL‐10 in both the liver and biliary fluid. Reduced IL‐2 in the liver suggests compromised T cell activation, while elevated IL‐10 in the biliary fluid may indicate an anti‐inflammatory response. This disparity demonstrates compartmentalised immune activation in PSCs.

## 4 Discussion

This systematic review explored differences of immune signatures in patients with PSC (with and without concomitant IBD) and in those with IBD alone (UC and CD). Comparative analysis of immune cell subsets and cytokine profiles in these two groups revealed that the immune profile in the patients with PSC is at least in part different from that in patients with UC or CD alone (Figure 3).

In PSC (with and without IBD), there is an obvious increase in various immune cell types and associated pro‐inflammatory mediators, along with a decrease in anti‐inflammatory cytokines compared to control. These occurred in the liver, biliary fluid, and systemic circulation, indicating a widespread pro‐inflammatory environment contributing to chronic liver inflammation and associated fibrosis. Another important fact to highlight is that the levels of immune activation in these body parts are different. Therefore, it is expected that the circulatory cells will affect the local immune responses by the resident immune cells, which could potentially influence disease progression, treatment approaches, and responses [66].

The clinical association of IBD, especially with UC, further complicates the immunological landscape of PSC. In our systematic review, a considerable number of patients with PSC also had concomitant IBD, and no distinctive immune profile of the patients with PSC vs PSC‐IBD has been investigated in the available studies, making it challenging to disentangle PSC‐specific immune signatures from those driven by co‐existing conditions. Despite these limitations, this systematic review highlights the immune profile that may be distinctive to PSC. For instance, Tregs play a crucial role in regulating inflammation, maintaining immune homeostasis, and establishing tolerance. They are generally present in similar numbers in the inflamed tissues compared to the non‐inflamed sections in patients with IBD [67]. In a systematic review including 22 studies, Jalalvand et al. found that the average ratio of Tregs in IBD compared with controls was slightly elevated. Specifically, patients with UC had a lower ratio, while those with CD exhibited a higher ratio [50].

While a decrease in Tregs is observed in patients with UC [24, 25, 34], those with PSC exhibit even lower levels of Tregs. It is well known that IL‐2, through its receptor CD25, is involved in the generation, maturation, and function of Tregs, and a low dose of IL‐2 showed promise as a therapeutic agent in inflammatory and autoimmune diseases, including hepatitis C virus‐induced vasculitis [68, 69, 70]. Therefore, decreased levels of Tregs within the PBMCs, IL‐2 and IL‐10 in the liver may orchestrate the dysregulated function of Tregs in PSC. Furthermore, CD28 plays a critical role in inducing the expression of cell surface negative regulators of T cell function and promoting the production of IL‐2. This mechanism provides protection against autoimmune diseases, pathogens, and graft rejection [71]. Here, we observed that the CD28‐ T cell population was increased in both patients with IBD and PSC [37, 72], further confirming the dysregulation in the immune regulatory function in PSC. These findings suggest that Tregs could serve as a potential pathologic marker, emphasising the importance of developing Treg‐specific targeted therapies to boost natural immune defences instead of relying on conventional immunosuppressive agents [73]. Moreover, it has been proposed that gut microbiota, dietary carbohydrates, proteins, fats, and vitamin D play an important role in the differentiation and function of Tregs in the thymus [74, 75, 76]. Therefore, gut microbiome profiling may identify another potential avenue of PSC pathogenesis. Additionally, a proper diet that boosts the development of Tregs [75], and their anti‐inflammatory function could potentially alleviate immune dysregulation in PSC and IBD [77].

Collectively, the data show that in patients with IBD, there is a consistent augmented Th17 immune response and an increase of the related cytokines IL‐17 and IL‐22 [78]. The consensus from the literature included in this review confirmed this association to be found in PSC patients as well. Interestingly, elevated levels of IL‐17‐producing cells have been observed in various acute and chronic liver diseases, including those with established fibrosis and cirrhosis [79, 80], without concomitant gastrointestinal inflammation. This indicates that an increase in Th17 cells could manifest independently of intestinal inflammation or through a synergistic pathologic action of PSC and concomitant IBD. The IL‐17 receptor is highly expressed in the liver tissue. Therefore, binding of IL‐17 with its receptor likely plays a crucial role in promoting immune cell infiltration and production of inflammatory cytokine IL‐6, as well as markers of fibrosis such as collagen, matrix metalloproteinase‐9, tissue inhibitor of metalloproteinase‐1 and 2. These further contribute to the development of chronic inflammation and fibrosis in the liver [80]. Recent studies suggest that alteration in intestinal microbiota could promote IL‐17 production by the intrahepatic γδ+ T cells and cause cholestatic liver disease [38]. Therefore, modulating the gut microbiome has been proposed as a promising strategy to develop new and personalised therapies to treat PSC [15]. We also observed an increase in β7+ gut‐homing T cell population in the liver tissue, which may also contribute to the inflammatory process in the liver of patients with PSC. However, these cells were reported to be commonly increased in chronic liver diseases, including PSC and proposed to be a characteristic feature of chronic inflammation [35]. With respect to this, two clinical trials have explored the treatment effects of targeting α4β7 integrin in PSC; however, the effects were minimal with regard to the biochemical parameters of liver function [81, 82]. Thus, more work is required to confirm that treatments targeting gut‐homing T‐cells have beneficial effects in PSCs.

IFN‐γ, another pro‐inflammatory cytokine expressed by both Th17 T cells [83] and γδ^+^ T cells [80], has been reported to play a crucial role in maintaining immune responses in the liver. It has been reported that genetic deletion of the *Ifng* gene reduced hepatotoxicity by suppressing CD8^+^ cytotoxic T cells and NK cells. This also promoted the shifting of macrophages to a more anti‐inflammatory phenotype to ameliorate liver fibrosis [84]. A recent report by Kellerer et al. showed that the expression of TNF‐related apoptosis‐inducing ligand (TRAIL) and granzyme B was increased in CD8^+^ T cells and NK cells in PSCs. Granzyme B promoted apoptosis and fibrosis, whereas TRAIL normalised the cytotoxic immune responses and attenuated liver injury and fibrosis, as shown in different genetically modified in vivo models of PSC [85]. Since dysbiosis has been proposed to be one of the root causes of PSC pathogenesis [15], Seidel et al. reported that CD8^+^T cells primed in the gut lymphoid tissues could migrate to the liver and induce cholangitis [86]. NK cells were also found to promote gut microbiota‐driven immune responses in the liver, exacerbating tissue injury [87]. Therefore, the increase of CD8^+^ T, NK, and Th17 cells in the PSC liver suggests their involvement in the inflammatory process through upregulation of granzyme B, IL‐17 and IFN‐γ, where restoring dysbiosis could mitigate immune‐driven liver injury [15]. Along with IL‐17, we also found that the level of Th17‐derived IL‐22 was elevated in the systemic circulation and in the liver. IL‐22 is a critical cytokine reported to have the capability to suppress immune activation in the mucosal epithelium by regulating major histocompatibility complex II [88] and promote epithelial cell proliferation and wound healing in mucosal inflammation [89]. In the liver, IL‐22 was found to activate JAK/STAT3 and mitogen activated protein kinase (MAPK) pathways and was reviewed to reduce inflammation and fibrosis in chronic liver disease [90, 91]. Recently, Sajiir et al. developed a fusion protein of IL‐22 that successfully targeted the liver in different in vivo models of metabolic dysfunction‐associated non‐alcoholic steatohepatitis and significantly reduced chronic liver inflammation [92]. These results indicate that IL‐22 could be a potential anti‐inflammatory therapeutic entity that can target liver as well as mucosal inflammation to treat PSC with or without concomitant IBD.

Among other observed pro‐inflammatory cytokines and chemokines, IL‐8 or CXCL8 is a chemoattractant for neutrophils and macrophages and is reported to cause acute inflammation in the liver [93]. Although the levels of IL‐8 were found to be elevated in chronic liver diseases, its precise role in the pathogenesis of PSC remains to be fully elucidated. TNF‐α is well‐known to activate MAPK and NFκB pathways, leading to systemic inflammation [94]. In the liver, this cytokine was reported to promote the progression from steatosis to non‐alcoholic steatohepatitis (NASH) by upregulating the expression of hepatocyte‐derived IL‐8 and chemokine CXCL1 to recruit neutrophils [95, 96, 97]. Hwang et al. showed IL‐22 to suppress CXCL1 that could ameliorate NASH [95]. In addition, the elevated levels of IL‐10 in both systemic circulation and bile, as well as the upregulation of IL‐22 in the systemic circulation indicate that the PSC patients possess an insufficient and parallel anti‐inflammatory initiative aimed at compensating inflammation in the liver. This also highlights that the delicate balance between immune overactivation and potential immune regulation in PSC is needed to be restored. Although, several biologics targeting macrophage‐associated cytokines such as TNF‐α and IL‐6 have shown excellence in UC and CD, they are not effective enough to treat or worsen PSC pathogenesis [16]. Therefore, alternative approaches including targeting Tregs, Th17 cells, macrophages, and the associated cytokines could facilitate immune balance and a safer successful management of PSC with or without IBD.

### 4.1 Limitations of the Study

We found variations in patient demographics, experimental methodologies, and sample size. Considering the variety of studies included in this review, the average quality score does not reflect different methodologies they used to identify immune signatures and does not explain the heterogeneity of the findings. Though the majority of articles clearly mentioned their aims and study limitations, provided patient demographics, and performed statistical analysis, inclusion and exclusion criteria were not clearly specified in 11 out of 23 studies. Only a few studies noted if the patients were receiving medication during sampling. The absence of consistent immune marker testing meant we could not combine studies into a meta‐analysis, and indeed many of the findings were contested across studies and some were from sole studies. While we used an unvalidated quality assessment scale as per Burns et al. [22] and supported by systematic reviews on tools for in vitro studies [23], a validated scale might provide a more robust evaluation of study quality. Additionally, 9 out of 23 articles involved only ≤ 10 PSC subjects, which reduced the power of their reported outcomes. Furthermore, none of these studies differentiated the immune profile in patients with PSC and PSC‐IBD to facilitate identification of PSC‐specific immune characteristics independent of IBD influence. The analysis also incorporated several articles with low‐quality scores due to limited data availability.

### 4.2 Conclusion and Future Directions

This systematic review demonstrated that the immune responses in PSC are organ‐specific, with obvious differences between liver tissue related immune activation and immune markers identified in the systemic circulation. The observed immune dysregulation is also, at least in part, different from that seen in patients with IBD without PSC, especially in relation to Th17 responses and immune regulatory mechanisms. These explain why biological therapies effective for IBD may not translate into clinical benefit for PSC [16] and suggest that an alternate therapeutic target, such as gut‐liver immune axis might be beneficial to control immune activation in PSC and associated liver damage [15]. In addition, advanced technologies, including transcriptomics, may provide further insights into specific immune cell populations within gut‐liver axis offering novel therapeutic targets. These approaches could also identify prognostic biomarkers, facilitating early and personalised therapeutic interventions.

## Author Contributions

M.M. and G.H. conceived and designed the study. M.M., A.S., and T.F. performed data acquisition. M.M. performed analysis, interpreted outcomes, and drafted manuscript. All authors made critical revisions to the manuscript. G.H. supervised the study and did funding acquisition.

## Conflicts of Interest

NJT: Prof. N. Talley. Disclosure: GutSee Ltd. (consulting microbiome, 2025), Brown University, Agency for Health Care Research and Quality (fiber and laxation) (2024), Rome Foundation (member gastroduodenal committee) (present), Biocodex (FD diagnostic tool) (present), Microba (consulting microbiome, 2025), Comvita Manuka Honey (FD trial consulting) (2025), BluMaiden (microbiome 2025) outside the submitted work. In addition, Dr. Talley has a patent Nepean Dyspepsia Index (NDI) 1998, a patent Licensing Questionnaires Talley Bowel Disease Questionnaire licensed to Mayo/Talley, “Diagnostic marker for functional gastrointestinal disorders” Australian Provisional Patent Application 2021901692, “Methods and compositions for treating age‐related neurodegenerative disease associated with dysbiosis” US Application No. 63/537,725. Financial support: Dr. Talley is supported by funding from the National Health and Medical Research Council (NHMRC) to the Center for Research Excellence in Digestive Health, and he holds an NHMRC Investigator grant.

GH: Prof. Gerald Holtmann received unrestricted educational support from the Falk Foundation. Research support was provided via the Princess Alexandra Hospital, Brisbane by GI. Therapies Pty Ltd., Takeda Development Center Asia, Pty Ltd., Eli Lilly Australia Pty Ltd., F. Hoffmann‐La Roche Ltd., MedImmune Ltd., Celgene Pty Ltd., Celgene International II Sarl, Gilead Sciences Pty Ltd., Quintiles Pty Ltd., Vital Food Processors Ltd., Datapharm Australia Pty. Ltd. Commonwealth Laboratories, Pty Ltd., Prometheus Laboratories, FALK GmbH & Co KG, Nestle Pty Ltd., Mylan, and Allergan (prior to acquisition by AbbVie Inc.). Dr. Holtmann is also a patent holder for a biopsy device to take aseptic biopsies (US 20150320407 A1).

All other authors declare no conflicts of interest.

## Supporting information


Supporting Information S1

## Data Availability

The data underlying this article will be shared on reasonable request to the corresponding author.
