# Supplementary material for: Immune Activation in Primary Sclerosing Cholangitis: A Systematic Review and Comparative Analysis With Inflammatory Bowel Diseases
Source: United European Gastroenterol J. 2025 Sep 30;13(9):1740–53. doi: 10.1002/ueg2.70115 (PMC12605952; doi:10.1002/ueg2.70115)
Supplement: Supplementary file 1 — Supporting Information S1 [file UEG2-13-1740-s001.docx]

**Supplementary materials**

**Supplementary figure *1:*** *PRISMA flow diagram showing the process of literature search and study selection.*

**Supplementary table 1:** Systematic review search strategy.

| **#** | **Search strategy** |
| --- | --- |
| 1 | "Sclerosing cholangitis"[ti] "primary sclerosing cholangitis"[tiab] OR "PSC"[ti] OR cholangitis, sclerosing[mh] |
| 2 | Cholangitis[mh] AND sclerosis[mh] |
| 3 | "Biliary cholangitis"[ti] |
| 4 | #1 OR #2 NOT #3 |
| 5 | Inflammation[ti] OR cytokine*[ti] OR "liver inflammation"[tiab] OR "liver cytokine*"[tiab] OR "immune profile*"[tiab] OR "immune activation*"[tiab] OR "immune mediator*"[tiab] OR "TNF"[tiab] OR "IL-1B"[tiab] OR "IL-12"[tiab] OR "IL-18"[tiab] OR immunology[mh] OR immunopathogenesis[tiab] OR immunomodulation[tiab] OR "inflammatory mediators"[ti] OR "immune cell subsets"[tiab] |
| 6 | #4 AND #5 |
|  |  |
|  |  |
|  |  |
|  |  |

**Supplementary table 2:** Immune findings reported in the literature on UC and CD.

| **Results** | **Number of studies** | **Average quality score (/20)** | **References** |
| --- | --- | --- | --- |
| **Circulating immune cells in patients with UC** | | | |
| No change of CD4+ T cells | 1 | 14 | Kosoy et al. 2021, Cell Mol Gastroenterol Hepatol [[99](#_ENREF_99)] |
| Increased Th17 cells | 1 | 14 | Xue et al. 2019, Medicine [[100](#_ENREF_100)] |
| Increased Tfh cells | 2 | 14 | Xue et al. 2019, Medicine [[100](#_ENREF_100)]  Long et al. 2020, Front Immunol [[39](#_ENREF_39)] |
| Decreased CD25+ cells | 1 | 13 | Maul et al. 2005, Gastroenterology [[41](#_ENREF_41)] |
| No change of CD25+ cells | 1 | 14 | De Tena et al. 2004, J Clin Immunol [[40](#_ENREF_40)] |
| Increased γδ+ T cells | 1 | 12 | Giacomelli et al. 1994, Clin Exp Immunol [[43](#_ENREF_43)] |
| Increased NK cells | 1 | 14 | Kosoy et al. 2021, Cell Mol Gastroenterol Hepatol [[99](#_ENREF_99)] |
| Decreased β7+ cells | 2 | 11.5 | Meenan et al. 1997, Gut [[101](#_ENREF_101)]  Fischer et al. 2016, Gut [[102](#_ENREF_102)] |
| No change of β7+ cells | 1 | 14 | Gamliel et al. 2020, Clin Exp Gastroenterol [[103](#_ENREF_103)] |
| Decreased CCR6+ cells | 1 | 14 | Long et al. 2020, Front Immunol [[39](#_ENREF_39)] |
| **Circulating cytokines and other factors in patients with UC** | | | |
| Increased IL-1β | 4 | 14.5 | Fukushima et al. 1995, Dig Dis Sci [[104](#_ENREF_104)]  Coburn et al. 2013, PLoS One [[50](#_ENREF_50)]  Szkaradkiewicz et al. 2009, Arch Immunol Ther Exp [[105](#_ENREF_105)]  Tan et al. 2022, Cell Mol Biol [[106](#_ENREF_106)] |
| Decreased IL-2 | 2 | 14 | Garcia de Tena et al. 2006, J Clin Immunol [[107](#_ENREF_107)]  Fukushima et al. 1995, Dig Dis Sci [[104](#_ENREF_104)] |
| Increased IL-2 | 2 | 15.5 | Coburn et al. 2013, PLoS One [[50](#_ENREF_50)]  Tan et al. 2022, Cell Mol Biol [[106](#_ENREF_106)] |
| Increased IL-4 | 1 | 14 | Garcia de Tena et al. 2006, J Clin Immunol [[107](#_ENREF_107)] |
| No change of IL-4 | 1 | 17 | Coburn et al. 2013, PLoS One [[50](#_ENREF_50)] |
| Decreased IL-5 | 1 | 17 | Coburn et al. 2013, PLoS One [[50](#_ENREF_50)] |
| No change of IL-6 | 2 | 15.5 | Garcia de Tena et al. 2006, J Clin Immunol [[107](#_ENREF_107)]  Coburn et al. 2013, PLoS One [[50](#_ENREF_50)] |
| Increased IL-6 | 2 | 13.5 | Szkaradkiewicz et al. 2009, Arch Immunol Ther Exp [[105](#_ENREF_105)]  Tan et al. 2022, Cell Mol Biol [[106](#_ENREF_106)] |
| Increased IL-7 | 1 | 17 | Coburn et al. 2013, PLoS One [[50](#_ENREF_50)] |
| Increased IL-8 | 1 | 13 | Szkaradkiewicz et al. 2009, Arch Immunol Ther Exp [[105](#_ENREF_105)] |
| No change of IL-8 | 1 | 17 | Coburn et al. 2013, PLoS One [[50](#_ENREF_50)] |
| Increased IL-10 | 2 | 15 | Coburn et al. 2013, PLoS One [[50](#_ENREF_50)]  Szkaradkiewicz et al. 2009, Arch Immunol Ther Exp [[105](#_ENREF_105)] |
| No change of IL-10 | 1 | 14 | Garcia de Tena et al. 2006, J Clin Immunol [[107](#_ENREF_107)] |
| Increased IL-12p70 | 1 | 17 | Coburn et al. 2013, PLoS One [[50](#_ENREF_50)] |
| No change of IL-13 | 1 | 17 | Coburn et al. 2013, PLoS One [[50](#_ENREF_50)] |
| Increased IL-17A | 2 | 15.5 | Krawiec et al. 2020, Sci Rep [[108](#_ENREF_108)]  Abdul-Hussein et al. 2021, Iraqi J Sci [[109](#_ENREF_109)] |
| Increased IL-22 | 1 | 16 | Arj et al. 2018, Iran J Allergy Asthma Immunol [[110](#_ENREF_110)] |
| Decreased IL-22 | 1 | 16 | Sakemi et al. 2020, Cytokine [[52](#_ENREF_52)] |
| No change of IFN-γ | 2 | 15.5 | Garcia de Tena et al. 2006, J Clin Immunol [[107](#_ENREF_107)]  Coburn et al. 2013, PLoS One [[50](#_ENREF_50)] |
| Increased IFN-γ | 1 | 16 | Hameed et al. 2023, J Interferon Cytokine Res [[111](#_ENREF_111)] |
| Increased TNF-α | 3 | 15.3 | Coburn et al. 2013, PLoS One [[50](#_ENREF_50)]  Hameed et al. 2023, J Interferon Cytokine Res [[111](#_ENREF_111)]  Szkaradkiewicz et al. 2009, Arch Immunol Ther Exp [[105](#_ENREF_105)] |
| Increased CCL4 / MIP-1β | 1 | 17 | Coburn et al. 2013, PLoS One [[50](#_ENREF_50)] |
| Increased CCL11 / Eotaxin-1 | 2 | 15 | Coburn et al. 2013, PLoS One [[50](#_ENREF_50)]  Manousou et al. 2010, Clin Exp Immunol [[53](#_ENREF_53)] |
| Increased CCL22 / MDC | 1 | 17 | Coburn et al. 2013, PLoS One [[50](#_ENREF_50)] |
| Increased CCL24 / Eotaxin-2 | 1 | 13 | Manousou et al. 2010, Clin Exp Immunol [[53](#_ENREF_53)] |
| Increased CCL26 / Eotaxin-3 | 2 | 15 | Coburn et al. 2013, PLoS One [[50](#_ENREF_50)]  Manousou et al. 2010, Clin Exp Immunol [[53](#_ENREF_53)] |
| No change of CXCL10 / IP10 | 1 | 17 | Coburn et al. 2013, PLoS One [[50](#_ENREF_50)] |
| Increased CD40 | 2 | 12.5 | Ludwiczek et al. 2003, Int J Colorectal Dis [[54](#_ENREF_54)]  Sawada-Hase et al. 2000, Am J Gastroenterol [[112](#_ENREF_112)] |
| No change of CD40 | 1 | 17 | Coburn et al. 2013, PLoS One [[50](#_ENREF_50)] |
| **Tissue resident immune cells in patients with UC** | | | |
| Increased CD3+ T cells | 1 | 15 | Røyset et al. 2023, J Pathol Clin Res [[113](#_ENREF_113)] |
| No change of CD3+ T cells | 2 | 14 | Uguccioni et al. 1999, Am J Pathol [[114](#_ENREF_114)]  Smids et al. 2018, J Crohns Colitis [[57](#_ENREF_57)] |
| Increased CD4+ T cells | 1 | 15 | Smids et al. 2018, J Crohns Colitis [[57](#_ENREF_57)] |
| Increased CD8+ T cells | 1 | 15 | Corridoni et al. 2020, Nat Med [[115](#_ENREF_115)] |
| Decreased CD8+ T cells | 1 | 15 | Smids et al. 2018, J Crohns Colitis [[57](#_ENREF_57)] |
| Increased Th17 cells | 1 | 14 | Carrasco et al. 2016, J Crohns Colitis [[116](#_ENREF_116)] |
| Decreased γδ+ T cells | 1 | 9 | Lee et al. 1997, Korean J Intern Med [[59](#_ENREF_59)] |
| Increased Macrophage | 1 | 14 | Magnusson et al. 2016, Mucosal Immunol [[58](#_ENREF_58)] |
| Decreased Macrophage | 1 | 13 | Uguccioni et al. 1999, Am J Pathol [[114](#_ENREF_114)] |
| Decreased DC | 1 | 14 | Magnusson et al. 2016, Mucosal Immunol [[58](#_ENREF_58)] |
| Decreased β7+ cells | 1 | 9 | Meenan et al. 1997, Gut [[101](#_ENREF_101)] |
| Increased β7+ cells | 2 | 13.5 | Fischer et al. 2016, Gut [[102](#_ENREF_102)]  Souza et al. 1999, Gut [[65](#_ENREF_65)] |
| Increased B cells | 1 | 14 | Uzzan et al. 2022, Nat Med [[117](#_ENREF_117)] |
| Increased CD28- cells | 1 | 12 | Kobayashi et al. 2007, Inflamm Bowel Dis [[72](#_ENREF_72)] |
| **Tissue cytokines and other factors in patients with UC** | | | |
| Increased IL-1β | 4 | 13.5 | Coburn et al. 2013, PLoS One [[50](#_ENREF_50)]  Fukushima et al. 1995, Dig Dis Sci [[104](#_ENREF_104)]  Ligumsky et al. 1990, Gut [[63](#_ENREF_63)]  Onken et al. 2008, Clin Immunol [[64](#_ENREF_64)] |
| Increased IL-2 | 1 | 17 | Coburn et al. 2013, PLoS One [[50](#_ENREF_50)] |
| Decreased IL-2 | 1 | 14 | Fukushima et al. 1995, Dig Dis Sci [[104](#_ENREF_104)] |
| Increased IL-4 | 2 | 15.5 | Coburn et al. 2013, PLoS One [[50](#_ENREF_50)]  Tan et al. 2022, Cell Mol Biol [[106](#_ENREF_106)] |
| Decreased IL-4 | 1 | 13 | Fuss et al. 1996, J Immunol [[118](#_ENREF_118)] |
| Increased IL-6 | 4 | 14 | Coburn et al. 2013, PLoS One [[50](#_ENREF_50)]  Tan et al. 2022, Cell Mol Biol [[106](#_ENREF_106)]  Onken et al. 2008, Clin Immunol [[64](#_ENREF_64)]  Iboshi et al. 2017, J Gastroenterol [[119](#_ENREF_119)] |
| Increased IL-8 | 4 | 13.3 | Coburn et al. 2013, PLoS One [[50](#_ENREF_50)]  Uguccioni et al. 1999, Am J Pathol [[114](#_ENREF_114)]  Bruno et al. 2015, Dig Dis Sci [[120](#_ENREF_120)]  Brand et al. 2006, Am J Physiol Gastrointest Liver Physiol [[121](#_ENREF_121)] |
| Increased IL-10 | 2 | 15.5 | Coburn et al. 2013, PLoS One [[50](#_ENREF_50)]  Carrasco et al. 2022, Immun Inflamm Dis [[62](#_ENREF_62)] |
| Increased IL-17A | 4 | 14.3 | Coburn et al. 2013, PLoS One [[50](#_ENREF_50)]  Iboshi et al. 2017, J Gastroenterol [[119](#_ENREF_119)]  Nielsen et al. 2003, Scand J Gastroenterol [[122](#_ENREF_122)]  Dobre et al. 2018, J Immunol Res [[123](#_ENREF_123)] |
| Increased IFN-γ | 4 | 14 | Coburn et al. 2013, PLoS One [[50](#_ENREF_50)]  Iboshi et al. 2017, J Gastroenterol [[119](#_ENREF_119)]  Onken et al. 2008, Clin Immunol [[64](#_ENREF_64)]  Dobre et al. 2018, J Immunol Res [[123](#_ENREF_123)] |
| Increased TNF-α | 4 | 14 | Coburn et al. 2013, PLoS One [[50](#_ENREF_50)]  Iboshi et al. 2017, J Gastroenterol [[119](#_ENREF_119)]  Onken et al. 2008, Clin Immunol [[64](#_ENREF_64)]  Dobre et al. 2018, J Immunol Res [[123](#_ENREF_123)] |
| Increased CCL25 | 3 | 13 | Dobre et al. 2018, J Immunol Res [[123](#_ENREF_123)]  Trivedi et al. 2016, J Autoimmun [[124](#_ENREF_124)]  Zhu et al. 2014, PLoS One [[125](#_ENREF_125)] |
| **Circulating immune cells in patients with CD** | | | |
| No change of CD4+ T cells | 2 | 13 | Kosoy et al. 2021, Cell Mol Gastroenterol Hepatol [[99](#_ENREF_99)]  Holland et al. 2008, Pediatr Res [[126](#_ENREF_126)] |
| Decreased Th1 cells | 1 | 12 | Holland et al. 2008, Pediatr Res [[126](#_ENREF_126)] |
| Increase of Th17 cells | 2 | 11.5 | Kleinschek et al. 2009, J Exp Med [[127](#_ENREF_127)],  Dige et al. 2013, J Crohns Colitis [[128](#_ENREF_128)] |
| Increased Treg cells | 1 | 14 | Jalalvand et al. 2023, Int Immunopharmacol [[42](#_ENREF_42)] |
| Decreased CD25+ cells | 1 | 13 | Maul et al. 2005, Gastroenterology [[41](#_ENREF_41)] |
| No change of CD25+ cells | 1 | 14 | De Tena et al. 2004, J Clin Immunol [[40](#_ENREF_40)] |
| Increased γδ+ T cells | 1 | 12 | Giacomelli et al. 1994, Clin Exp Immunol [[43](#_ENREF_43)] |
| Increased NK cells | 1 | 14 | Kosoy et al. 2021, Cell Mol Gastroenterol Hepatol [[99](#_ENREF_99)] |
| Increased β7+ cells | 1 | 12 | Gamliel et al. 2020, Clin Exp Gastroenterol [[103](#_ENREF_103)] |
| Decreased β7+ cells | 2 | 11.5 | Meenan et al. 1997, Gut [[101](#_ENREF_101)]  Fischer et al. 2016, Gut [[102](#_ENREF_102)] |
| **Circulating cytokines and other factors in patients with CD** | | | |
| Increased IL-1β | 3 | 14.3 | Fukushima et al. 1995, Dig Dis Sci [[104](#_ENREF_104)]  Szkaradkiewicz et al. 2009, Arch Immunol Ther Exp [[105](#_ENREF_105)]  Tatsuki et al. 2020, Sci Rep [[51](#_ENREF_51)] |
| Increased IL-2 | 2 | 14 | Garcia de Tena et al. 2006, J Clin Immunol [[107](#_ENREF_107)]  Fukushima et al. 1995, Dig Dis Sci [[104](#_ENREF_104)] |
| Decreased IL-2 | 1 | 16 | Tatsuki et al. 2020, Sci Rep [[51](#_ENREF_51)] |
| Increased IL-4 | 1 | 16 | Tatsuki et al. 2020, Sci Rep [[51](#_ENREF_51)] |
| No change of IL-4 | 2 | 15 | Garcia de Tena et al. 2006, J Clin Immunol [[107](#_ENREF_107)]  Cho et al. 2018, J Korean Med Sci [[129](#_ENREF_129)] |
| Increased IL-5 | 2 | 15 | Tatsuki et al. 2020, Sci Rep [[51](#_ENREF_51)]  Scoville et al. 2019, Sci Rep [[130](#_ENREF_130)] |
| Increased IL-6 | 2 | 15 | Garcia de Tena et al. 2006, J Clin Immunol [[107](#_ENREF_107)]  Tatsuki et al. 2020, Sci Rep [[51](#_ENREF_51)] |
| Decreased IL-6 | 1 | 13 | Szkaradkiewicz et al. 2009, Arch Immunol Ther Exp [[105](#_ENREF_105)] |
| Increased IL-7 | 1 | 16 | Tatsuki et al. 2020, Sci Rep [[51](#_ENREF_51)] |
| Decreased IL-7 | 1 | 8 | Andreu-Ballester et al. 2013, Inflamm Bowel Dis [[131](#_ENREF_131)] |
| Increased IL-8 | 2 | 14.5 | Tatsuki et al. 2020, Sci Rep [[51](#_ENREF_51)]  Szkaradkiewicz et al. 2009, Arch Immunol Ther Exp [[105](#_ENREF_105)] |
| Increased IL-10 | 3 | 14.3 | Garcia de Tena et al. 2006, J Clin Immunol [[107](#_ENREF_107)]  Tatsuki et al. 2020, Sci Rep [[51](#_ENREF_51)]  Szkaradkiewicz et al. 2009, Arch Immunol Ther Exp [[105](#_ENREF_105)] |
| Increased IL-12p70 | 1 | 16 | Tatsuki et al. 2020, Sci Rep [[51](#_ENREF_51)] |
| Increased IL-13 | 1 | 16 | Tatsuki et al. 2020, Sci Rep [[51](#_ENREF_51)] |
| Increased IL-17A | 2 | 16 | Tatsuki et al. 2020, Sci Rep [[51](#_ENREF_51)]  Cho et al. 2018, J Korean Med Sci [[129](#_ENREF_129)] |
| Increased IL-22 | 2 | 15 | Cho et al. 2018, J Korean Med Sci [[129](#_ENREF_129)]  Schmechel et al. 2008, Inflamm Bowel Dis [[132](#_ENREF_132)] |
| Decreased IL-22 | 1 | 16 | Sakemi et al. 2020, Cytokine [[52](#_ENREF_52)] |
| Increased IFN-γ | 2 | 15 | Garcia de Tena et al. 2006, J Clin Immunol [[107](#_ENREF_107)]  Hameed et al. 2023, J Interferon Cytokine Res [[111](#_ENREF_111)] |
| Decreased IFN-γ | 1 | 16 | Tatsuki et al. 2020, Sci Rep [[51](#_ENREF_51)] |
| Increased TNF-α | 4 | 14.8 | Garcia de Tena et al. 2006, J Clin Immunol [[107](#_ENREF_107)]  Tatsuki et al. 2020, Sci Rep [[51](#_ENREF_51)]  Hameed et al. 2023, J Interferon Cytokine Res [[111](#_ENREF_111)]  Szkaradkiewicz et al. 2009, Arch Immunol Ther Exp [[105](#_ENREF_105)] |
| Increased CCL11 / Eotaxin-1 | 1 | 13 | Manousou et al. 2010, Clin Exp Immunol [[53](#_ENREF_53)] |
| No change of CCL22 / MDC | 1 | 10 | Galli et al. 2000, Eur J Immunol [[133](#_ENREF_133)] |
| Increased CCL24 / Eotaxin-2 | 1 | 13 | Manousou et al. 2010, Clin Exp Immunol [[53](#_ENREF_53)] |
| Increased CCL26 / Eotaxin-3 | 1 | 10 | Manousou et al. 2010, Clin Exp Immunol [[53](#_ENREF_53)] |
| Increased CD40 | 2 | 12.5 | Ludwiczek et al. 2003, Int J Colorectal Dis [[54](#_ENREF_54)]  Sawada-Hase et al. 2000, Am J Gastroenterol [[112](#_ENREF_112)] |
| **Tissue resident immune cells in patients with CD** | | | |
| Increased CD3+ T cells | 1 | 15 | Røyset et al. 2023, J Pathol Clin Res [[113](#_ENREF_113)] |
| Decreased CD3+ T cells | 1 | 15 | Smids et al. 2018, J Crohns Colitis [[57](#_ENREF_57)] |
| Increased CD4+ T cells | 2 | 12 | Smids et al. 2018, J Crohns Colitis [[57](#_ENREF_57)]  Humphreys et al. 2024, J Cell Mol Med [[60](#_ENREF_60)] |
| Decreased CD8+ T cells | 2 | 14 | Smids et al. 2018, J Crohns Colitis [[57](#_ENREF_57)]  Jaeger et al. 2021, Nat Commun [[134](#_ENREF_134)] |
| Increased Th17 cells | 1 | 14 | Carrasco et al. 2016, J Crohns Colitis [[116](#_ENREF_116)] |
| Decreased γδ+ T cells | 1 | 9 | Lee et al. 1997, Korean J Intern Med [[59](#_ENREF_59)] |
| Increased Macrophage | 1 | 14 | Magnusson et al. 2016, Mucosal Immunol [[58](#_ENREF_58)] |
| Decreased DC | 1 | 14 | Magnusson et al. 2016, Mucosal Immunol [[58](#_ENREF_58)] |
| Decreased β7+ cells | 1 | 9 | Meenan et al. 1997, Gut [[101](#_ENREF_101)] |
| Increased β7+ cells | 2 | 13.5 | Fischer et al. 2016, Gut [[102](#_ENREF_102)]  Souza et al. 1999, Gut [[65](#_ENREF_65)] |
| Increased B cells | 1 | 8.5 | Humphreys et al. 2024, J Cell Mol Med [[60](#_ENREF_60)]  Shen et al. 2023, Clin Exp Med [[135](#_ENREF_135)] |
| Increased CD28- cells | 1 | 12 | Kobayashi et al. 2007, Inflamm Bowel Dis [[72](#_ENREF_72)] |
| **Tissue cytokines and other factors in patients with CD** | | | |
| Increased IL-1β | 4 | 12 | Fukushima et al. 1995, Dig Dis Sci [[104](#_ENREF_104)]  Ligumsky et al. 1990, Gut [[63](#_ENREF_63)]  Onken et al. 2008, Clin Immunol [[64](#_ENREF_64)]  Reimund et al. 1996, Gut [[136](#_ENREF_136)] |
| Increased IL-2 | 1 | 14 | Fukushima et al. 1995, Dig Dis Sci [[104](#_ENREF_104)] |
| Increased IL-4 | 1 | 11 | Akiho et al. 2005, Am J Physiol Gastrointest Liver Physiol [[137](#_ENREF_137)] |
| Increased IL-6 | 2 | 13 | Onken et al. 2008, Clin Immunol [[64](#_ENREF_64)]  Ng et al. 2011, Inflamm Bowel Dis [[61](#_ENREF_61)] |
| Increased IL-8 | 3 | 11.3 | Brandt et al. 2020, Clin Exp Immunol [[138](#_ENREF_138)]  Bruno et al. 2015, Dig Dis Sci [[120](#_ENREF_120)]  Brand et al. 2006, Am J Physiol Gastrointest Liver Physiol [[121](#_ENREF_121)] |
| Increased IL-10 | 1 | 14 | Carrasco et al. 2022, Immun Inflamm Dis [[62](#_ENREF_62)] |
| No change of IL-10 | 1 | 14 | Ng et al. 2011, Inflamm Bowel Dis [[61](#_ENREF_61)] |
| Increased IL-17A | 2 | 13.5 | Nielsen et al. 2003, Scand J Gastroenterol [[122](#_ENREF_122)]  Dobre et al. 2018, J Immunol Res [[123](#_ENREF_123)] |
| Increased IFN-γ | 1 | 12 | Onken et al. 2008, Clin Immunol [[64](#_ENREF_64)] |
| Increased TNF-α | 2 | 11.5 | Onken et al. 2008, Clin Immunol [[64](#_ENREF_64)]  Reimund et al. 1996, Gut [[136](#_ENREF_136)] |

**Supplementary table 3:** Quality assessment scoring system.

| **Criteria** | **0** | **1** | **2** |
| --- | --- | --- | --- |
| **Are the aims of the study clearly stated?** | Aims not stated | Some aims stated clearly/all aims stated unclearly/not in abstract | All aims stated clearly & included in abstract |
| **Are the inclusion & exclusion criteria for participants included?** | No mention of participant criteria | Only either inclusion or exclusion criteria included | Both inclusion & exclusion criteria included |
| **Are appropriate controls used?** | No description of controls provided | Used non-PSC non-IBD diseased controls | Used healthy controls |
| **Are participant demographic characteristics included?** | No reference to participant characteristics | Some reference to participant characteristics | Detailed references to participant characteristics |
| **Are any comorbidities acknowledged & controlled for?** | Controlling for comorbidities not mentioned | Comorbidities mentioned but not controlled/some comorbidities mentioned | Comorbidities mentioned & controlled for |
| **Was ethical approval obtained & acknowledged?** | No reference to ethical approval | Ethical approval from unnamed body | Ethical approval gained from a named body |
| **Were power calculations performed?** | No mention of power calculations performed | Power calculation attempted | Study numbers match power calculation |
| **Are statistics appropriate?** | No statistics/inappropriate statistics | Appropriate statistics but no justification given | Appropriate statistics used with justification |
| **Is there any indication that only certain immune markers were reported because they had significant p-values?** | Only statistically significant results were reported | Only data with statistically significant and close to significant p-values were reported | All results were reported irrespective to p-values |
| **Are limitations of the study acknowledged?** | No limitations acknowledged | Some but not all limitations acknowledged/acknowledgement of limitations unclear | Limitations acknowledged |
| Total score: ____/20 | | | |

**Supplementary table 4:** Immune findings reported in the literature on PSC.

| **Results** | **Number of studies** | **Average quality score** | **References** |
| --- | --- | --- | --- |
| **Circulating immune cells** | | | |
| Increased CD3+ T cells | 2 | 11.5 | Adam et al. 2018, Hepatol Commun [[30](#_ENREF_30)]  Bo et al. 2001, Gut [[37](#_ENREF_37)] |
| Increased CD4+ T cells | 1 | 16 | Adam et al. 2018, Hepatol Commun [[30](#_ENREF_30)] |
| Decreased Th1 cells | 2 | 15.5 | Adam et al. 2018, Hepatol Commun [[30](#_ENREF_30)]  Gwela et al. 2017, J Crohns Colitis [[32](#_ENREF_32)] |
| No change of Th2 cells | 1 | 16 | Adam et al. 2018, Hepatol Commun [[30](#_ENREF_30)] |
| Increased Th17 cells | 5 | 15.4 | Adam et al. 2018, Hepatol Commun [[30](#_ENREF_30)]  Gwela et al. 2017, J Crohns Colitis [[32](#_ENREF_32)]  Dold et al. 2023, Clin Transl Gastroenterol [[31](#_ENREF_31)]  Katt et al. 2013, Hepatology [[33](#_ENREF_33)]  Poch et al. 2021, J Hepatol [[34](#_ENREF_34)] |
| Increased Tfh cells | 1 | 16 | Adam et al. 2018, Hepatol Commun [[30](#_ENREF_30)] |
| Increased Treg cells | 1 | 16 | Adam et al. 2018, Hepatol Commun [[30](#_ENREF_30)] |
| Decreased Treg cells | 2 | 14 | Abarbanel et al. 2013, J Clin Immunol [[35](#_ENREF_35)]  Sebode et al. 2014, J Hepatol [[36](#_ENREF_36)] |
| No change of CD25+ cells | 1 | 7 | Bo et al. 2001, Gut [[37](#_ENREF_37)] |
| Increased γδ+ T cells | 1 | 10 | Martins et al. 1996, Hepatology [[25](#_ENREF_25)] |
| Increased NK cells | 1 | 7 | Bo et al. 2001, Gut [[37](#_ENREF_37)] |
| Decreased NK cells | 1 | 12 | Liu et al. 2022, Front Immunol [[38](#_ENREF_38)] |
| Increased β7+ cells | 1 | 15 | Gwela et al. 2017, J Crohns Colitis [[32](#_ENREF_32)] |
| Increased CCR6+ cells | 1 | 15 | Gwela et al. 2017, J Crohns Colitis [[32](#_ENREF_32)] |
| **Circulating cytokines and other factors** | | | |
| Increased IL-1β | 1 | 12 | Landi et al. 2014, J Interferon Cytokine Res [[48](#_ENREF_48)] |
| Increased IL-2 | 2 | 12.5 | Landi et al. 2014, J Interferon Cytokine Res [[48](#_ENREF_48)]  Broome et al. 1998, Dis Colon Rectum [[44](#_ENREF_44)] |
| Decreased IL-4 | 2 | 12.5 | Landi et al. 2014, J Interferon Cytokine Res [[48](#_ENREF_48)]  Broome et al. 1998, Dis Colon Rectum [[44](#_ENREF_44)] |
| Increased IL-5 | 2 | 14 | Landi et al. 2014, J Interferon Cytokine Res [[48](#_ENREF_48)]  Lampinen et al. 2018, J Leukoc Biol [[46](#_ENREF_46)] |
| Increased IL-6 | 3 | 14.3 | Landi et al. 2014, J Interferon Cytokine Res [[48](#_ENREF_48)]  Dold et al. 2023, Clin Transl Gastroenterol [[31](#_ENREF_31)]  Zweers et al. 2016, Liver Int [[26](#_ENREF_26)] |
| Increased IL-7 | 1 | 12 | Landi et al. 2014, J Interferon Cytokine Res [[48](#_ENREF_48)] |
| Increased IL-8 | 3 | 11.3 | Landi et al. 2014, J Interferon Cytokine Res [[48](#_ENREF_48)]  Zweers et al. 2016, Liver Int [[26](#_ENREF_26)]  Bansal et al. 1997, Autoimmunity [[29](#_ENREF_29)] |
| Increased IL-10 | 2 | 10.5 | Landi et al. 2014, J Interferon Cytokine Res [[48](#_ENREF_48)]  Bansal et al. 1997, Autoimmunity [[29](#_ENREF_29)] |
| Decreased IL-12p70 | 1 | 12 | Landi et al. 2014, J Interferon Cytokine Res [[48](#_ENREF_48)] |
| Increased IL-13 | 2 | 14 | Landi et al. 2014, J Interferon Cytokine Res [[48](#_ENREF_48)]  Lampinen et al. 2018, J Leukoc Biol [[46](#_ENREF_46)] |
| Increased IL-17A | 1 | 18 | Dold et al. 2023, Clin Transl Gastroenterol [[31](#_ENREF_31)] |
| Increased IL-22 | 1 | 14 | Poch et al. 2021, J Hepatol [[34](#_ENREF_34)] |
| No change of IL-18R1 | 1 | 16 | Lampinen et al. 2019, J Crohns Colitis [[47](#_ENREF_47)] |
| Increased IFN-γ | 2 | 15 | Landi et al. 2014, J Interferon Cytokine Res [[48](#_ENREF_48)]  Dold et al. 2023, Clin Transl Gastroenterol [[31](#_ENREF_31)] |
| Increased TNF-α | 1 | 13 | Zweers et al. 2016, Liver Int [[26](#_ENREF_26)] |
| Decreased TNF-α | 1 | 12 | Landi et al. 2014, J Interferon Cytokine Res [[48](#_ENREF_48)] |
| Increased CCL4 / MIP-1β | 1 | 12 | Landi et al. 2014, J Interferon Cytokine Res [[48](#_ENREF_48)] |
| Increased CCL11 / Eotaxin-1 | 1 | 16 | Lampinen et al. 2018, J Leukoc Biol [[46](#_ENREF_46)] |
| No change of CCL13 / MCP-4 | 1 | 12 | Landi et al. 2014, J Interferon Cytokine Res [[48](#_ENREF_48)] |
| No change of CCL17 / TARC | 1 | 12 | Landi et al. 2014, J Interferon Cytokine Res [[48](#_ENREF_48)] |
| Decreased CCL22 / MDC | 1 | 12 | Landi et al. 2014, J Interferon Cytokine Res [[48](#_ENREF_48)] |
| Increased CCL24 / Eotaxin-2 | 1 | 16 | Lampinen et al. 2018, J Leukoc Biol [[46](#_ENREF_46)] |
| Increased CCL26 / Eotaxin-3 | 1 | 16 | Lampinen et al. 2018, J Leukoc Biol [[46](#_ENREF_46)] |
| Increased CXCL9 | 1 | 12 | Landi et al. 2014, J Interferon Cytokine Res [[48](#_ENREF_48)] |
| Increased CXCL10 / IP10 | 2 | 10.5 | Landi et al. 2014, J Interferon Cytokine Res [[48](#_ENREF_48)]  Langeneckert et al. 2019, Eur J Immunol [[49](#_ENREF_49)] |
| Increased CXCL11 | 1 | 9 | Langeneckert et al. 2019, Eur J Immunol [[49](#_ENREF_49)] |
| Increased sCD14 | 1 | 16 | Dhillon et al. 2019, Liver Int [[45](#_ENREF_45)] |
| Increased sCD23 | 1 | 9 | Bansal et al. 1997, Autoimmunity [[29](#_ENREF_29)] |
| No change of CD40 | 1 | 16 | Lampinen et al. 2019, J Crohns Colitis [[47](#_ENREF_47)] |
| **Cytokines in biliary fluid** | | | |
| Increased IL-2  Increased IL-4  Increased IL-6  Increased IL-10  Increased IL-17A  Increased IFN-γ | 1 | 12 | Zhou et al. 2020, Eur J Gastroenterol Hepatol [[28](#_ENREF_28)] |
| Increased IL-8 | 1 | 13 | Zweers et al. 2016, Liver Int [[26](#_ENREF_26)] |
| **Liver tissue-resident immune cell** | | | |
| Decreased CD3+ T cells | 2 | 7.5 | Bo et al. 2001, Gut [[37](#_ENREF_37)]  Hashimoto et al. 1993, Mayo Clin Proc [[24](#_ENREF_24)] |
| Decreased CD4+ T cells | 1 | 8 | Hashimoto et al. 1993, Mayo Clin Proc [[24](#_ENREF_24)] |
| Increased CD8+ T cells | 1 | 8 | Hashimoto et al. 1993, Mayo Clin Proc [[24](#_ENREF_24)] |
| Increased Th17 cells | 1 | 5 | Tedesco et al. 2018, Gastroenterology [[27](#_ENREF_27)] |
| Increased CD25+ T cells  Decreased CD25+ T cells | 1  1 | 8  7 | Hashimoto et al. 1993, Mayo Clin Proc [[24](#_ENREF_24)]  Bo et al. 2001, Gut [[37](#_ENREF_37)] |
| Increased γδ+ T cells  Decreased γδ+ T cells | 1  1 | 10  5 | Martins et al. 1996, Hepatology [[25](#_ENREF_25)]  Tedesco et al. 2018, Gastroenterology [[27](#_ENREF_27)] |
| Increased Macrophages | 1 | 8 | Hashimoto et al. 1993, Mayo Clin Proc [[24](#_ENREF_24)] |
| No change of Dendritic cells | 1 | 8 | Hashimoto et al. 1993, Mayo Clin Proc [[24](#_ENREF_24)] |
| Increased NK cells  Decreased NK cells | 1  1 | 8  7 | Hashimoto et al. 1993, Mayo Clin Proc [[24](#_ENREF_24)]  Bo et al. 2001, Gut [[37](#_ENREF_37)] |
| Increased β7+ cells | 1 | 10 | Graham et al. 2022, Hepatology [[55](#_ENREF_55)] |
| Decreased B cells | 1 | 8 | Hashimoto et al. 1993, Mayo Clin Proc [[24](#_ENREF_24)] |
| Increased CD28- T cells | 1 |  | Liaskou et al. 2014, Gastroenterology [[56](#_ENREF_56)] |
| **Liver tissue cytokines** | | | |
| Increased IL-1β  Decreased IL-2  Decreased IL-10  Increased TNF-α | 1 | 7 | Bo et al. 2001, Gut [[37](#_ENREF_37)] |
| Increased CCL25 | 1 | 10 | Graham et al. 2022, Hepatology [[55](#_ENREF_55)] |

**ADDITIONAL REFERENCES**

99. Kosoy R et al. Deep Analysis of the Peripheral Immune System in IBD Reveals New Insight in Disease Subtyping and Response to Monotherapy or Combination Therapy*.* Cell Mol Gastroenterol Hepatol 2021; 12: 599-632.

100. Xue GH et al. Aberrant alteration of follicular T helper cells in ulcerative colitis patients and its correlations with interleukin-21 and B cell subsets*.* Medicine 2019; 98.

101. Meenan J et al. Altered expression of alpha 4 beta 7, a gut homing integrin, by circulating and mucosal T cells in colonic mucosal inflammation*.* Gut 1997; 40: 241-6.

102. Fischer A et al. Differential effects of α4β7 and GPR15 on homing of effector and regulatory T cells from patients with UC to the inflamed gut in vivo*.* Gut 2016; 65: 1642-U290.

103. Gamliel A et al. Circulating alpha4beta7(+) Memory T Cells in Pediatric IBD Patients Express a Polyclonal T Cell Receptor Repertoire*.* Clin Exp Gastroenterol 2020; 13: 439-447.

104. Fukushima K, West G Fiocchi C. Adequacy of mucosal biopsies for evaluation of intestinal cytokine-specific mRNA. Comparative study of RT-PCR in biopsies and isolated cells from normal and inflamed intestine*.* Dig Dis Sci 1995; 40: 1498-505.

105. Szkaradkiewicz A et al. Proinflammatory cytokines and IL-10 in inflammatory bowel disease and colorectal cancer patients*.* Arch Immunol Ther Exp (Warsz) 2009; 57: 291-4.

106. Tan Y et al. Correlations of Inflammatory Cytokines in the Intestinal Mucosa, Serum Inflammation, Oxidative Stresses and Immune Changes with Vitamin Deficiency in Ulcerative Colitis Patients*.* Cell Mol Biol 2022; 68: 101-106.

107. Garcia de Tena J et al. Distinctive pattern of cytokine production and adhesion molecule expression in peripheral blood memory CD4+ T cells from patients with active Crohn's disease*.* J Clin Immunol 2006; 26: 233-42.

108. Krawiec P, Pac-Kozuchowska E. Serum interleukin 17A and interleukin 17F in children with inflammatory bowel disease*.* Sci Rep 2020; 10: 12617.

109. Abdul-Hussein SS et al. Roles of IL-17A and IL-23 in the pathogenesis of ulcerative colitis and Crohn’s disease*.* Iraqi J Sci 2021; 62: 2526-2535.

110. Arj A et al. The Correlation between the Numerical Status of Th22 Cells and Serum Level of IL-22 with Severity of Ulcerative Colitis*.* Iran J Allergy Asthma Immunol 2018; 17: 78-84.

111. Hameed NAA, Shaker OG Hasona NA. LINC00641/miR-378a and Their Cross-Talk with TNF-α/IFN-γ as Potential Biomarkers in Ulcerative Colitis and Crohn's Diseases*.* J Interferon Cytokine Res 2023; 43: 531-537.

112. Sawada-Hase N et al. An increased number of CD40-high monocytes in patients with Crohn's disease*.* Am J Gastroenterol 2000; 95: 1516-23.

113. Røyset ES et al. Deep learning-based image analysis reveals significant differences in the number and distribution of mucosal CD3 and γδ T cells between Crohn's disease and ulcerative colitis*.* J Pathol Clin Res 2023; 9: 18-31.

114. Uguccioni M et al. Increased expression of IP-10, IL-8, MCP-1, and MCP-3 in ulcerative colitis*.* Am J Pathol 1999; 155: 331-6.

115. Corridoni D et al. Single-cell atlas of colonic CD8(+) T cells in ulcerative colitis*.* Nat Med 2020; 26: 1480-1490.

116. Carrasco A et al. Regional Specialisation of T Cell Subsets and Apoptosis in the Human Gut Mucosa: Differences Between Ileum and Colon in Healthy Intestine and Inflammatory Bowel Diseases*.* J Crohns Colitis 2016; 10: 1042-1054.

117. Uzzan M et al. Ulcerative colitis is characterized by a plasmablast-skewed humoral response associated with disease activity*.* Nat Med 2022; 28: 766-+.

118. Fuss IJ et al. Disparate CD4+ lamina propria (LP) lymphokine secretion profiles in inflammatory bowel disease. Crohn's disease LP cells manifest increased secretion of IFN-gamma, whereas ulcerative colitis LP cells manifest increased secretion of IL-5*.* J Immunol 1996; 157: 1261-70.

119. Iboshi Y et al. Increased IL-17A/IL-17F expression ratio represents the key mucosal T helper/regulatory cell-related gene signature paralleling disease activity in ulcerative colitis*.* J Gastroenterol 2017; 52: 315-326.

120. Bruno ME et al. Correlation of Biomarker Expression in Colonic Mucosa with Disease Phenotype in Crohn's Disease and Ulcerative Colitis*.* Dig Dis Sci 2015; 60: 2976-84.

121. Brand S et al. IL-22 is increased in active Crohn's disease and promotes proinflammatory gene expression and intestinal epithelial cell migration*.* Am J Physiol Gastrointest Liver Physiol 2006; 290: G827-G838.

122. Nielsen OH et al. Upregulation of interleukin-12 and -17 in active inflammatory bowel disease*.* Scand J Gastroenterol 2003; 38: 180-5.

123. Dobre M et al. Differential Intestinal Mucosa Transcriptomic Biomarkers for Crohn's Disease and Ulcerative Colitis*.* J Immunol Res 2018; 2018: 9208274.

124. Trivedi PJ et al. Intestinal CCL25 expression is increased in colitis and correlates with inflammatory activity*.* J Autoimmun 2016; 68: 98-104.

125. Zhu S et al. CCL25/CCR9 interactions regulate the function of iNKT cells in oxazolone-induced colitis in mice*.* PLoS One 2014; 9: e100167.

126. Holland N et al. Reduced intracellular T-Helper 1 interferon-gamma in blood of newly diagnosed children with Crohn's disease and age-related changes in Th1/Th2 cytokine profiles*.* Pediatr Res 2008; 63: 257-262.

127. Kleinschek MA et al. Circulating and gut-resident human Th17 cells express CD161 and promote intestinal inflammation*.* J Exp Med 2009; 206: 525-34.

128. Dige A et al. Increased levels of circulating Th17 cells in quiescent versus active Crohn's disease*.* J Crohns Colitis 2013; 7: 248-55.

129. Cho J et al. Mucosal Immunity Related to FOXP3(+) Regulatory T Cells, Th17 Cells and Cytokines in Pediatric Inflammatory Bowel Disease*.* J Korean Med Sci 2018; 33: e336.

130. Scoville EA et al. Serum Polyunsaturated Fatty Acids Correlate with Serum Cytokines and Clinical Disease Activity in Crohn's Disease*.* Sci Rep 2019; 9: 2882.

131. Andreu-Ballester JC et al. Deficit of interleukin-7 in serum of patients with Crohn's disease*.* Inflamm Bowel Dis 2013; 19: E30-1.

132. Schmechel S et al. Linking genetic susceptibility to Crohn's disease with Th17 cell function: IL-22 serum levels are increased in Crohn's disease and correlate with disease activity and IL23R genotype status*.* Inflamm Bowel Dis 2008; 14: 204-12.

133. Galli G et al. Macrophage-derived chemokine production by activated human T cells in vitro and in vivo: preferential association with the production of type 2 cytokines*.* Eur J Immunol 2000; 30: 204-10.

134. Jaeger N et al. Single-cell analyses of Crohn's disease tissues reveal intestinal intraepithelial T cells heterogeneity and altered subset distributions*.* Nat Commun 2021; 12: 1921.

135. Shen X et al. Identification of antigen-presentation related B cells as a key player in Crohn's disease using single-cell dissecting, hdWGCNA, and deep learning*.* Clin Exp Med 2023; 23: 5255-5267.

136. Reimund JM et al. Increased production of tumour necrosis factor-alpha interleukin-1 beta, and interleukin-6 by morphologically normal intestinal biopsies from patients with Crohn's disease*.* Gut 1996; 39: 684-9.

137. Akiho H et al. Interleukin-4- and -13-induced hypercontractility of human intestinal muscle cells-implication for motility changes in Crohn's disease*.* Am J Physiol Gastrointest Liver Physiol 2005; 288: G609-15.

138. Brandt E et al. Enhanced production of IL-8 in chronic but not in early ileal lesions of Crohn's disease (CD)*.* Clin Exp Immunol 2000; 122: 180-5.
